# Comparison Between Closed and Open Methods for Creating Pneumoperitoneum in Laparoscopic Cholecystectomy

**DOI:** 10.7759/cureus.35991

**Published:** 2023-03-10

**Authors:** Puneet K Agarwal, Jason Golmei, Richa Goyal, Ajeet P Maurya

**Affiliations:** 1 General Surgery, All India Institute of Medical Sciences Bhopal, Bhopal, IND; 2 Obstetrics and Gynecology Surgery, Dr. Agarwal Clinic, Jalesar, IND

**Keywords:** comparison, open, closed, methods, pneumoperitoneum

## Abstract

*Background:* To study the efficacy of closed and open methods for creating pneumoperitoneum in laparoscopic cholecystectomy by comparing the two in terms of their outcome and complication. *Study Design*: Single-centre, prospective, observational study. *Materials and study*: Purposive sampling method where the inclusion criteria were all patients with cholelithiasis who were advised and consented to laparoscopic cholecystectomy of age 18-70 years were included in the study group. Exclusion criteria include patients with a paraumbilical hernia, a history of upper abdominal surgery, uncontrolled systemic illness, and local skin infection. Sixty cases of cholelithiasis satisfying exclusion and inclusion criteria who underwent elective cholecystectomy during the study period were included. Thirty-one of these cases underwent the closed method, while in the remaining 29 patients open method was adopted. Cases in which pneumoperitoneum created by closed technique were grouped as group A and those by open technique as group B. Parameters comparing the safety and efficacy of the two methods were studied. The parameters were access time, gas leak, visceral injury, vascular injury, need for conversion, umbilical port site hematoma, umbilical port site infection, and hernia. Patients were assessed on the first postoperative day, the seventh postoperative day, and then two months after surgery. Some follow-ups were done telephonically. *Results*: Out of 60 patients, 31 underwent the closed method, while 29 underwent the open method. Minor complications like gas leak during the procedure was observed more in the open method. The mean access time in the open-method group was less than in the closed-method group. Other complications like visceral injury, vascular injury, need for conversion, umbilical port site hematoma, umbilical port site infection, and hernia were not observed in either group during the allocated follow-up period in the study. *Conclusion*: Open technique for pneumoperitoneum is as safe and effective as the closed technique.

## Introduction

The word laparoscopy originated from the Greek word Laparo - which means abdomen, and scopion- meaning to examine. Laparoscopy is the art of evaluating the abdominal cavity and its contents. This is achieved by creating a pneumoperitoneum where the abdominal cavity is insufflated with gas to establish sufficient space and visualize the abdominal contents using an illuminated telescope field [1]. Currently, laparoscopy is widely used in the practice of medicine for both diagnostic and therapeutic purposes. This minimally invasive approach has become the method of choice for treating many abdominal diseases that require surgery. However, laparoscopic procedures are not risk-free as laparoscopic entry is a blind procedure and presents its problem. The most crucial step of a laparoscopic procedure is the creation of a pneumoperitoneum. Complications related to laparoscopic surgery are rare and commonly occur when accessing the peritoneal cavity [2]. Studies have shown the process of establishment of pneumoperitoneum to be associated with injuries related to the abdominal viscera and major blood vessels. At least 50% of these complications occur before the commencement of the surgery. This complication rate has remained unchanged over the last 25 years [3,4]. In a significant majority of patients, more than 50 % of all complications occur at the time of entry, and a great majority occur during the insertion of the primary umbilical trocar [5]. The incidence of vascular injuries in laparoscopy is 2 in 10,000 procedures, and 3.3 cases of serious complication associated with mortality per 1,00,000 cases of laparoscopy are known to occur [6,7]. There are two methods for creating pneumoperitoneum [8]. The classic closed technique (where Verres needle is used) and classic open technique (Hasson method, where Hasson’s cannula is used) [9,10] are the most common procedures to gain peritoneal access in laparoscopy. Pneumoperitoneum is conventionally induced by blind Verres needle insertion at the umbilicus, followed by blind trocar entry at the same site once pneumoperitoneum has been established. This blind primary access is the main challenge in the procedure. In the last three decades, rapid advances in laparoscopic surgery have made it invaluable to many surgical specialties, including general surgery. However, there remains no clear consensus as to which is an optimal method of entry into the peritoneal cavity [6].

## Materials and methods

This is a single-center, prospective, observational study approved by the Institutional Human and Ethical Committee (IHEC) conducted in a tertiary care hospital in Northern India. *Inclusion criteria: *all patients with cholelithiasis who were advised and consented to laparoscopic cholecystectomy aged 18-70 years. *Exclusion criteria*: patients with a paraumbilical hernia, a history of upper abdominal surgery, uncontrolled systemic illness, and local skin infection. Sixty patients satisfying inclusion and exclusion criteria underwent laparoscopic cholecystectomy in the general surgery department during this study period. Cases in which pneumoperitoneum was created by closed technique were grouped as group A, and those by open technique as group B. Thirty-one of these cases underwent the closed method. In contrast, in the remaining 29 patients open method was adopted. The method adopted for each patient was solely an observation from the surgeon's operative step. In group A patients, it was observed that either an infra- or supra-umbilical transverse incision was made through the skin to the subcutaneous tissue, and pneumoperitoneum was created with the help of a Veress needle. Peritoneal access was confirmed by applying aspiration and saline tests. With successful placement, the Veress needle was attached to the insufflator. CO2 in-sufflation started at a rate of 1 liter per minute and gradually increased to a higher flow rate until the pre-adjusted intrabdominal pressure reached 12 mmHg. The Veress needle was removed, and a 10 mm primary trocar was introduced, keeping the abdominal wall elevated. Trocar was then removed, followed by the insertion of the laparoscope into the cannula. Insufflator was then attached to the cannula, and the peritoneal cavity was inspected thoroughly for any injury inflicted. Time from incision to laparoscope insertion was recorded. Three more trocars were inserted under vision, and surgery was completed. Facial closure of port sites was performed with Port Vicryl (Ethicon) or Vicryl (Ethicon) No. 1, whereas skin closure was done with Ethilon (Ethicon) 2-0 RC. In group B, an infra-umbilical incision of 1-2 cm length was given through the skin to the subcutaneous tissue. The umbilical stalk was identified. The surrounding tissues were dissected using a strong Kocher's forceps, held, and lifted upwards. A cut was then made at its origin from the anterior abdominal wall. Thus, a rounded defect in the peritoneal cavity was created. The peritoneal breach dilated with the artery forceps. Any underlying adhesions were released with blunt dissection by the surgeon's finger with care. The margins were lifted using artery forceps and stay sutures with Vicryl (Ethicon) No 1; reverse cutting was placed on both sides of the defect. The stay sutures were held, and the 10 mm primary port cannula with a blunt obturator was introduced through the defect. The obturator was removed with the successful entry into the peritoneal cavity, and the cannula was connected to the insufflator. The laparoscope was then introduced into the cannula with adequate pneumoperitoneum established and the peritoneal cavity inspected. As in group A, the time from incision to laparoscope insertion was recorded. Three more trocars were inserted under vision, and surgery was completed. Closure of the umbilical port was performed by tying the stay sutures together. Parameters comparing the safety and efficacy of the two methods were studied. The parameters were access time, gas leak, visceral injury, vascular injury, need for conversion, umbilical port site hematoma, umbilical port site infection, and hernia. Patients were assessed on the first postoperative day, the seventh postoperative day, and then two months after surgery. Some of the follow-ups had to be done telephonically. *Access Time* was defined by the Time taken from the point of the first incision to the insertion of the laparoscope through the primary port cannula into the peritoneum. *Operating time* was defined as the time taken from the point of the first incision to the closure of the last remaining port. *Vascular injury* was defined by injury of the great vessels, namely, the abdominal aorta and inferior vena cava, during port establishment either by Veress needle or trocars. The *visceral injury *was defined by injury of the peritoneal cavity's visceral organs, including mesentery and the omentum, during port establishment either by Veress needle or trocars. *Postoperative vomiting *was defined as one or more episodes of vomiting within 48 hrs of surgery. *Postoperative urinary retention* was defined as the inability to void within 12 hrs of surgery. The port-site* hematoma* was defined as the presence of a hemorrhagic discharge or clot from the port-site wound within seven days of surgery. *Port-site infection* was defined by Sero-purulent and/or pus discharge from the port-site wound within seven days of surgery. *Port-site hernia* was defined as developing a hernia at the umbilical port site by the 30^th^ day of surgery or beyond.

*Statistical analysis* was carried out using statistical packages for SPSS Inc. Released 2007. SPSS for Windows, Version 16.0. Chicago, SPSS Inc. Continuous and categorical variables were expressed as mean ± SD and frequency (percentages). Student's t-test was applied to compare the two groups. Two-sided p-values are considered statistically significant at p<0.05.

## Results

Sixty patients fulfilling the inclusion and exclusion criteria who underwent elective laparoscopic cholecystectomy were included in the study. Out of 60 patients, 31 underwent laparoscopic cholecystectomy by the closed method, labelled as group A, and the remaining 29 underwent laparoscopic cholecystectomy by the open method, labelled as group B.

Gender distribution

Group A comprises 25 female patients (80.6%) and six male patients (19.4%). Group B comprises 20 female patients (69%) and nine male patients (31%).

**Figure 1:**
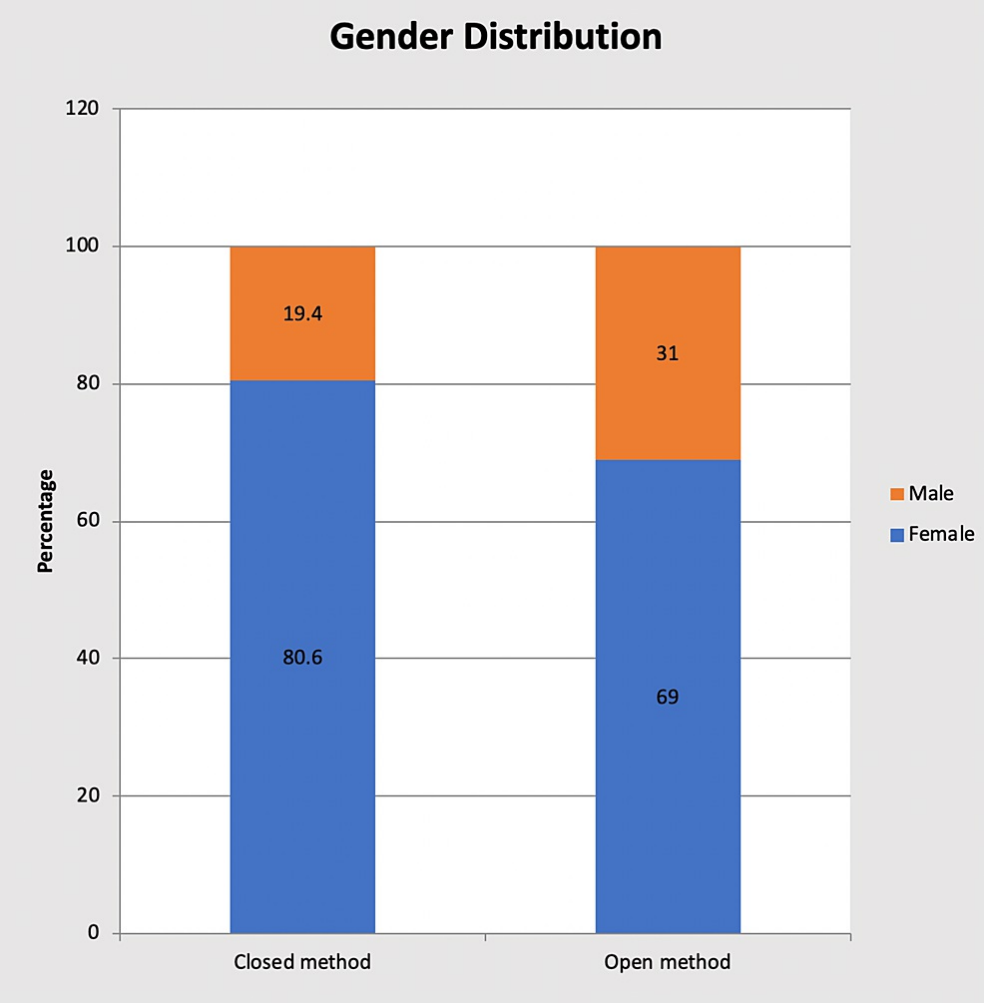
Bar chart showing gender distribution in each group

The study included 15 male and 45 female patients, making it a male: female ratio of 1:3 (Table 1 and Figure 1).

**Table 1:** Gender distribution

Sex	Closed Method (n=31)		Open Method (n=29)	
	Frequency	Percentage	Frequency	Percentage
Female	25	80.6%	20	69.0%
Male	6	19.4%	9	31.0%
Total	31	100.0%	29	100.0%

Age distribution 

In group A: There were four patients in the age group 18-31 years (12.9%), 14 patients in the age group 32-45 years (45.2%), and 13 patients in the age group 46-60 years (41.9%).

In group B: There were three patients in the age group 18-31 years (10.3%), 16 patients in the age group 32-45 years (55.2%), and 10 patients in the age group 46-60 years (34.5%).

**Figure 2:**
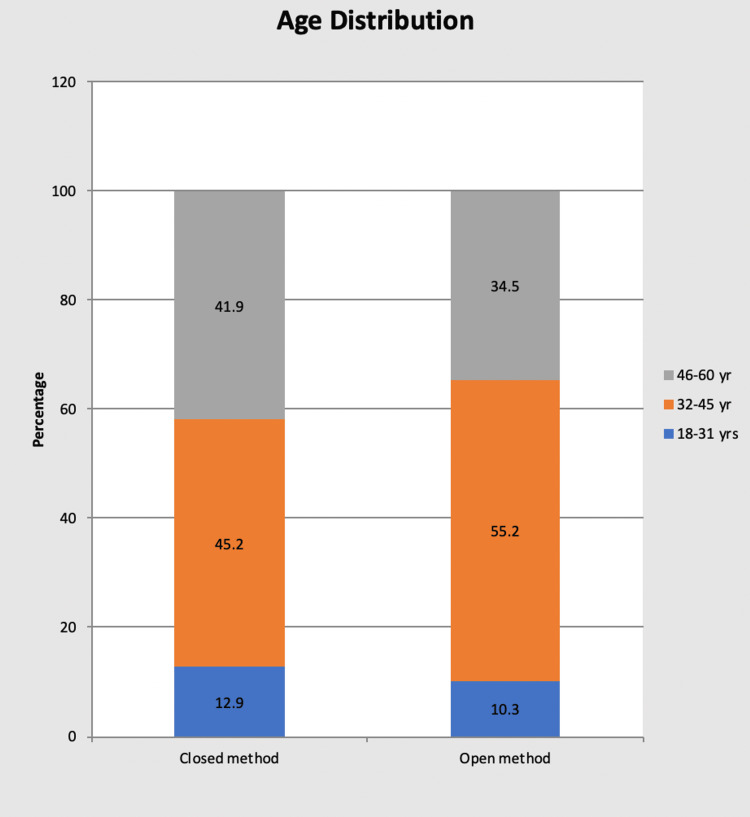
Bar chart showing age distribution in each group

A maximum of patients who underwent laparoscopic cholecystectomy by either method were seen in the age group of 32-45 years. 45.2% in group A and 55.2% in group B, respectively (Table 2 and Figure 2).

**Table 2:** Age distribution percentage (%)

Age in years	Closed method (n=31)		Open method (n=29)	
	Frequency	Percentage	Frequency	Percentage
18 -31	4	12.9%	3	10.3%
32-45	14	45.2%	16	55.2%
46-60	13	41.9%	10	34.5%
Total	31	100%	29	100%

Time taken for access

In group A: The time required for creating pneumoperitoneum ranged from 6 to 12 minutes. The largest number of patients found subjected to the access of pneumoperitoneum were in the time range of 6-10 minutes (28 patients). The mean time of induction of the pneumoperitoneum in this group was 9.16 minutes. The standard deviation calculated was 1.09 with a p-value of 0.049, considered significant.

**Figure 3:**
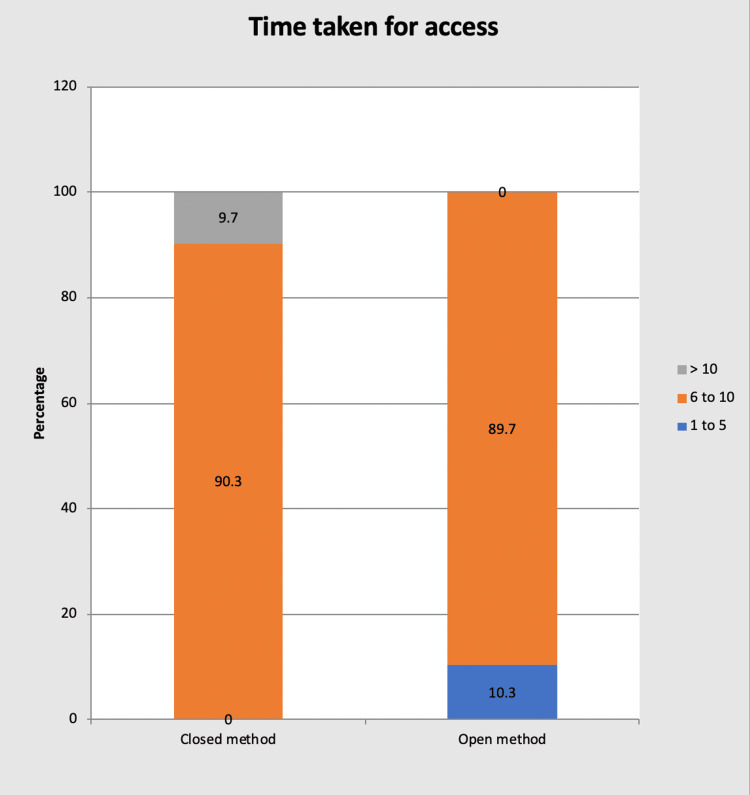
Bar chart showing the time taken for access in each group

In group B: The time required for creating pneumoperitoneum ranged from 5 to 10 minutes. The largest number of patients found subjected to the access of pneumoperitoneum were in the time range of 6-10 minutes (26 patients). The mean time of induction of the pneumoperitoneum in this group was 6.93 minutes. The standard deviation calculated was 0.99 with a p-value of 0.049, considered significant (sig) (Table 3 and Figure 3).

**Table 3:** Time taken for access Sig: Significant, HS: Highly significant

Time taken for access (in min)	Closed method (n=31)		Open method (n=29)		P-value
	Frequency	Percentage	Frequency	Percentage	
1-5	0	0%	3	10.3%	0.049(sig)
6-10	28	90.3%	26	89.7%	
>10	3	9.7%	0	0%	
Total	31	100%	29	100%	
Mean	9.16		6.93		<0.001 (HS)
SD	1.09		0.99		

In group A: Time is taken for access: Mean ± SD = 9.16 ± 1.09 and p-value of 0.049.

**Figure 4:**
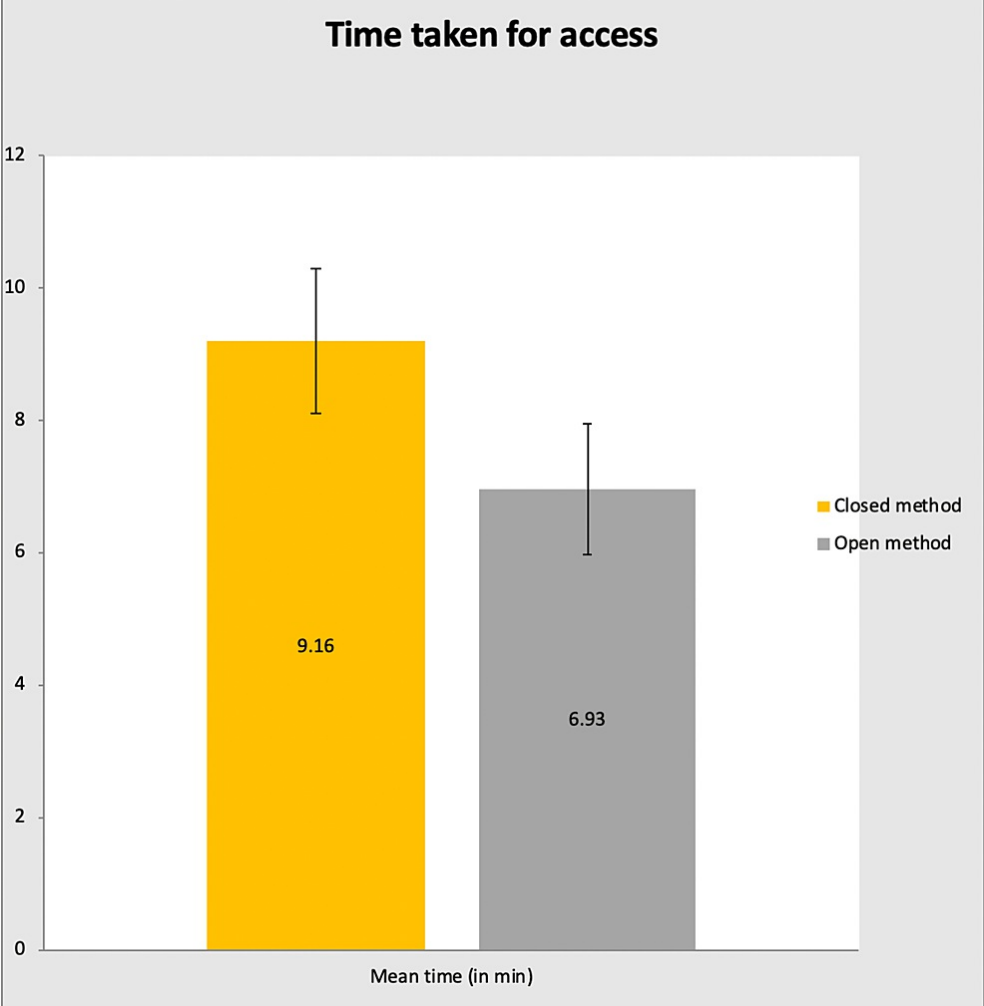
Bar chart showing the mean time taken for access in each group Min: Minutes

In group B: Time is taken for access: Mean ± SD = 6.93 ± 0.99 and p-value of 0.049. (Figure 4 shows the mean time taken for access).

Intra-operative complications

In group A: The gas leak was observed in three cases (9.7%).

However, intra-operative complications like visceral injury/vascular injury/need for conversion to open cholecystectomy were not observed.

**Figure 5:**
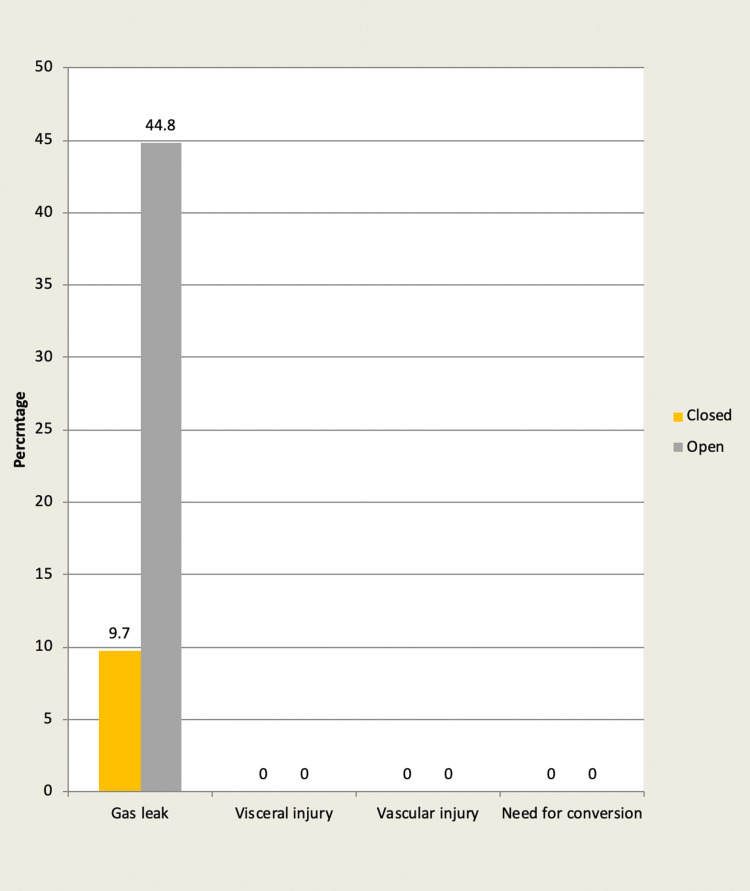
Bar chart showing the intra-operative complication encountered in each group

In group B: The gas leak was significant and observed in 13 cases (44.8%). P-value 0.002 significant. However, intra-operative complications like visceral injury/vascular injury/need for conversion to open cholecystectomy were not observed (Figure 5).

Postoperative complications

*Postoperative vomiting: *In group A, postoperative vomiting was observed in seven of the cases (22.6%). In group B, postoperative vomiting was observed in 10 cases (34.5%).

*Postoperative urinary retention: *In group A, postoperative urinary retention was observed in five patients (16.1%). In group B, postoperative urinary retention was observed in three patients (10.3%).

In either group: The p-value for post-operative vomiting is 0.155. The p-value for postoperative urinary retention is 0.274.

**Figure 6:**
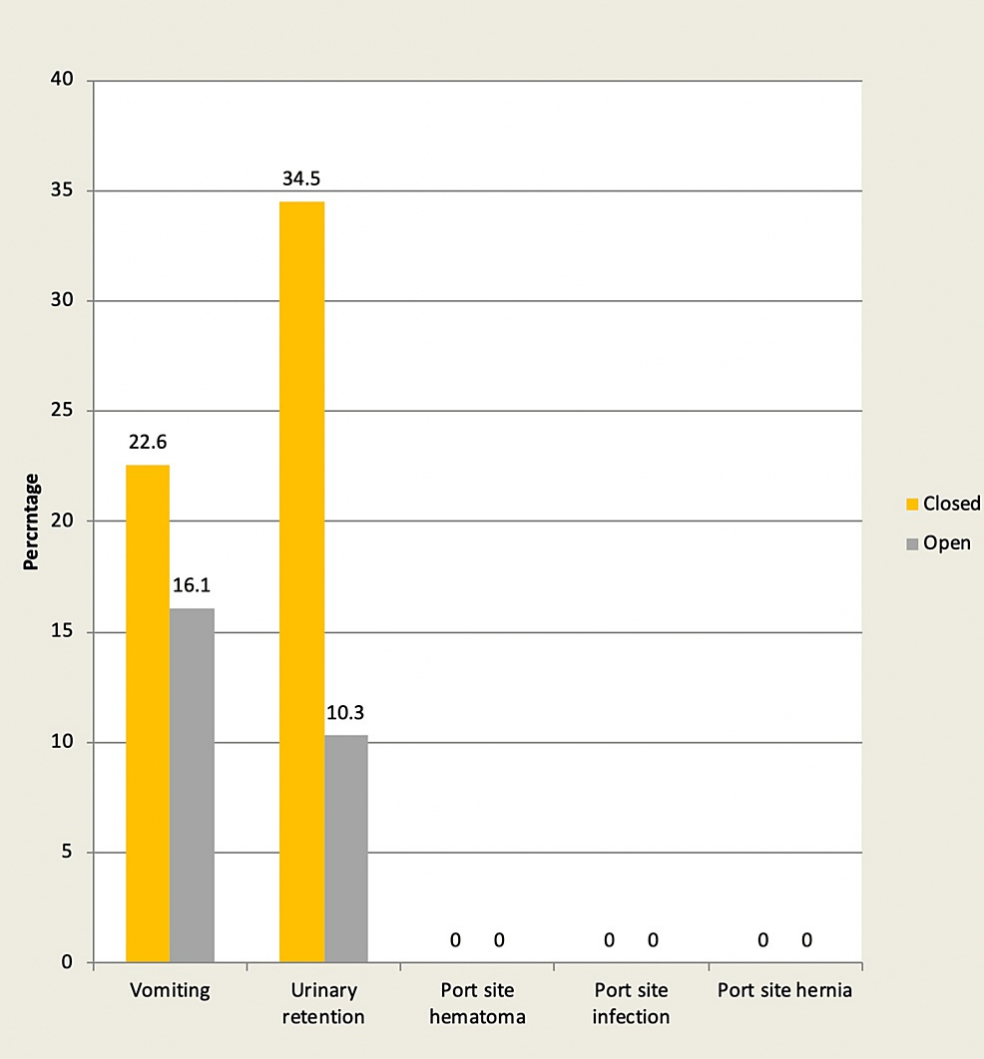
Bar chart showing post-operative complications in each group.

However, other complications like port site hematoma/port site infection and hernia were not observed in both groups (Figure 6).

Time required to close the wound, complete surgery, and hospital stay post-surgery 

In group A: The time required to close the wound ranged from 6-10 minutes, for which the mean ± SD is 7.06 ± 1.03. Total operating time ranged from 60-120 min, which means ± SD is 98.35 + 14.7. Duration of hospital stay in days ranged from 2-12 days, which means ± SD is 4.25 +2.46.

In group B: The time required to close the wound ranged from 5-15 minutes, for which the mean ± SD is 7.13+1.99. Total operating time ranged from 60-120 min, which means ± SD is 93.68+16.98. Duration of hospital stay in days ranged from 1-8 days, which means ± SD is 3.48+1.54.

In either group: The p-value for the time required to close the wound is 0.857, which is not significant. The p-value for total operating time is 0.263, which is not significant. The p-value for the duration of hospital stay is 0.148, which is not significant (Table 4).

**Table 4:** Time required to close the wound, complete surgery and hospital stay post-surgery Independent t test; p>0.05 not significant.

Variable	Closed method		Open method		
	Range	Mean±SD	Range	Mean±SD	P-value
Time required to close the wound (in min)	6-10	7.06 ±1.03	5-15	7.13±1.99	0.857
Total operating time (in min)	60-120	98.35±14.7	60-120	93.68±16.98	0.263
Hospital stay (in days)	2-12	4.25±2.46	1-8	3.48±1.54	0.148

**Figure 7:**
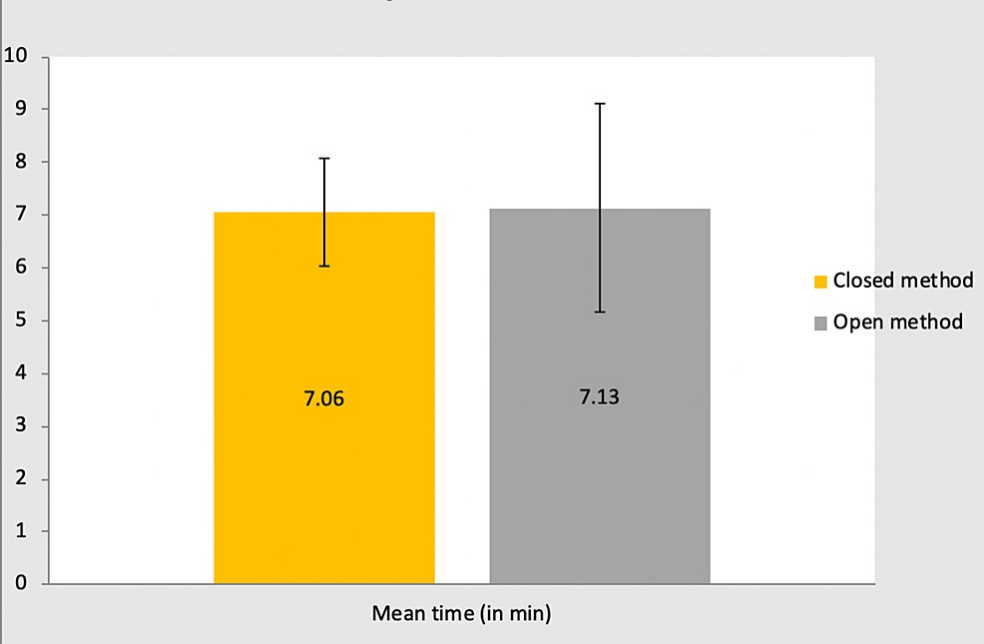
Bar chart showing the mean time to close the wound in each group

The mean time required to close the wound in group A was 7.06 minutes, and in group B was 7.13 minutes. However, it was observed to be statistically not significant (Figure 7).

**Figure 8:**
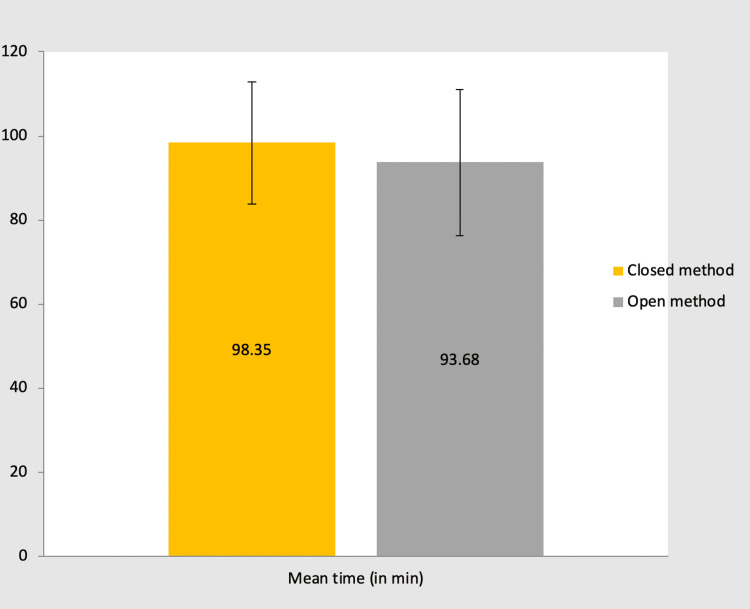
Bar chart showing mean time to complete the surgery (total operating time) in each group

The mean time required to complete the surgery (total operating time) in group A and group B was 98.35 and 93.68 minutes, respectively. However, it was observed to be statistically not significant (Figure 8).

**Figure 9:**
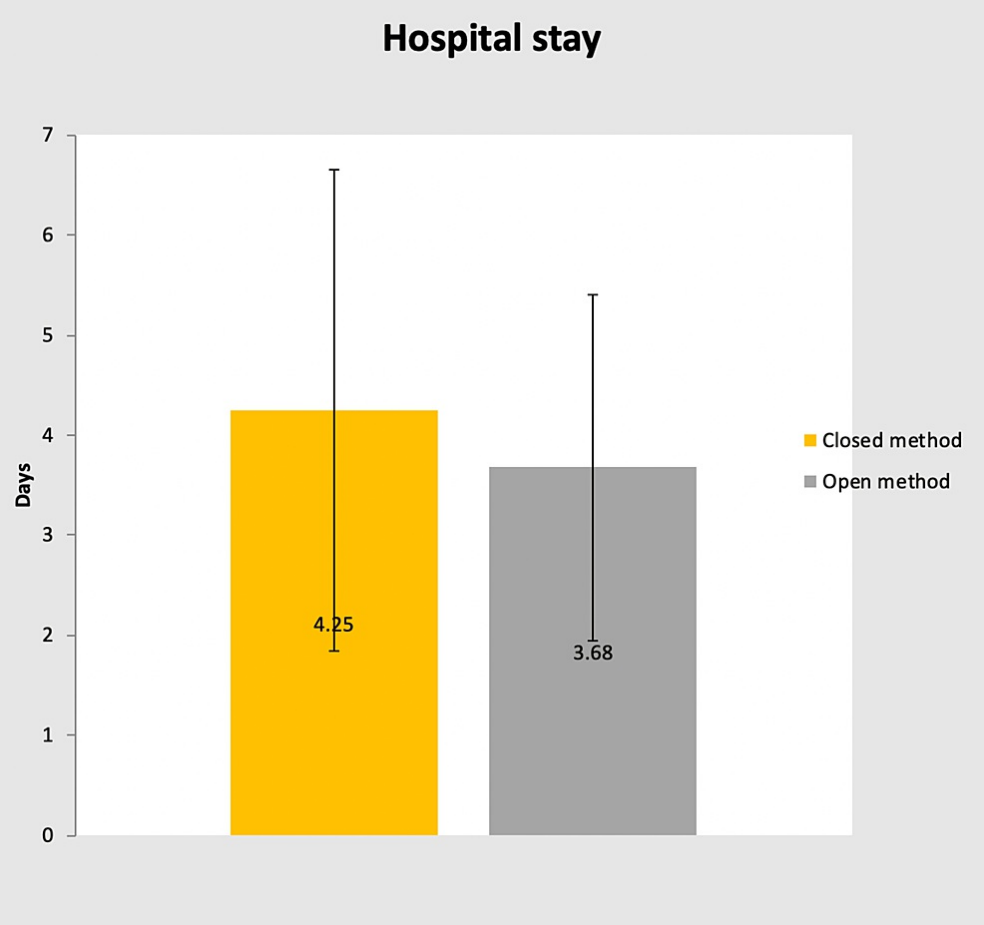
Bar chart showing mean hospital stay after surgery in each group

The mean duration of hospital stay post-surgery in groups A and B were 4.25 days and 3.68 days, respectively. However, it was observed to be statistically not significant (Figure 9). 

The data retrieved from this study suggest that both the closed and open methods for gaining access to the peritoneal cavity are safe. The open technique had a time advantage over the closed method in terms of access. Major complications like vascular and visceral injuries were not observed in any of the groups. Patients who underwent the closed method were subjected more to the risk of technique failure in the form of extraperitoneal insufflations, loss of space, and entry in the wrong place, although not considered part of the study variables, were also encountered a few times. Patients who underwent the open method had more chances of being subjected to a gas leak which could present a problem during the procedure.

## Discussion

Laparoscopic cholecystectomy is a minimally invasive surgical procedure, and since the early 1990s, open cholecystectomy has been replaced essentially by laparoscopic cholecystectomy [11]. Laparoscopic cholecystectomy has become the gold standard in treating gallstone disease [12]. However, laparoscopy is relatively new; therefore, it still arouses concerns and controversy, particularly in relation to the best method for the creation of the pneumoperitoneum. And the most important step in the laparoscopic procedure is the creation of the pneumoperitoneum. Iatrogenic injury related to laparoscopic procedures during access to the peritoneal cavity is still a problem faced by many surgeons [13]. The conventional closed pneumoperitoneum method involves blind entry into the peritoneal cavity using the Veress needle and yields the greatest risk of injury during this primary blind access. More than half of the complications arising from a laparoscopic procedure are observed to occur before the beginning of the actual surgery, that is, during the creation of pneumoperitoneum and trocar entry. The worst-case scenario for laparoscopic surgeons would be an iatrogenic injury to the great vessels or hollow viscus injury and eventually the need for conversion to open procedure. To prevent these unpredictable complications, other methods of pneumoperitoneum have been introduced over the last few decades, including the open method of pneumoperitoneum introduced by Harrith Hasson, optical trocars, direct trocar insertion, radically expanding trocars and the use of disposable shielded trocars [14-17]. Thus, the creation of pneumoperitoneum can be dealt with in many ways. However, the Veress needle and Hasson’s pneumoperitoneum method are the two most commonly used methods today [18]. The morbidity associated with creating pneumoperitoneum and primary trocar insertion is estimated to be < 1%; however, the incidence of complications like a vascular and visceral injury for both methods is unknown [19]. In this observational study, these methods were compared regarding the time required to induce pneumoperitoneum, the time needed to close the wounds, the total operating time, and complications associated with each method. Harrith Hasson introduced the open method of pneumoperitoneum in 1974. Although this method yields a lower risk of visceral or vascular injury, it did not gain wide acceptance as it was reported to be associated with a significant gas leak and time-consuming. The method was explicitly recommended for patients with previous upper abdominal surgery [18]. However, our study did not include patients with previous abdominal surgery.

The access time for creating pneumoperitoneum and inserting the camera port was 9.16 ± 1.09 minutes in group A (closed method), whereas, in group B (open method), it was 6.93 ± 0.99 minutes. Borgatta, in 1990, reported the meantime for pneumoperitoneum by closed method to be 130 seconds (2.2 minutes) [20]. Byron et al., in 1993, also said the mean access time for successful pneumoperitoneum in the closed method to be 5.9 ± 2.2 minutes [21]. A study by Ann-Cathrin Moberg published in a Scandinavian journal suggested that the blind Veress method of pneumoperitoneum requires 214-300 seconds (3.5-5 minutes) for abdominal cavity access [22]. Soomro et al., in 2004, reported that the mean time required for creating pneumoperitoneum with a Veress needle (closed method) was 5 minutes, and with the open method was 8 minutes [23]. Akbar et al. (2007) reported the mean time required for a successful pneumoperitoneum to be 9.17 minutes in the closed method and 8.11 minutes in the open method [12]. Angioli R et al. in 2013 also reported a mean access time of 212.4 seconds (3.54 minutes) in the closed group, whereas it was of 161.7 seconds (2.69 minutes) in the open group [24]. In 2014 Ilias Juneja et al. reported that the mean time required for access in the closed method was 2.83 minutes, and in the open method was 2.52 minutes [1]. Dr. Neet R. Chotai et al. reported in 2015 that the mean operative time for access in the closed method was 3.94 minutes, and in the open method was 5.12 minutes [6]. Tariq Nawaz et al. in 2016 reported that the mean time needed to create pneumoperitoneum with the closed technique was 4 ± 1 minutes and the open method was 5 ± 1 minutes, respectively (p-value = 0.000). Jamil et al. (2018) reported that the mean access time in the closed method was 6.58 ± 1.78 minutes, and in the open method was 5.49 ± 1.82 minutes [5].

In our study, 3 (9.7%) patients out of 31 in group A (closed method) developed gas leaks, whereas 13 (44.8%) cases of gas leaks out of 29 patients were observed in group B (open method). Similar findings have been reported with other workers as well, with the incidence of gas leaks leaning more towards the open method of pneumoperitoneum [1,3,5]. The gas leak is one factor that contributes to longer surgery duration, as it repeatedly interferes with the procedure (Table 5).

**Table 5:** Access time of pneumoperitoneum in both methods was reported in different studies

Studies	Access Time for Closed Method (in Minutes)	Access Time for Open Method (in Minutes)
Borgatta (1990) [20]	2.2	2
Byron (1993) [21]	5.9 ± 2.2	4.2 ± 1.3
Soomro (2004) [23]	5	8
Akbar et al. (2007) [12]	9.17	8.11
Angioli (2013) [24]	3.54	2.69
Ilias Juneja (2014) [1]	2.83	2.52
Chotai (2015) [6]	5.12 ± 2.5	3.94 ± 2.2
Tariq (2016) [3]	5 ± 1	4 ± 1
Jamil et al. (2018) [5]	6.58 ± 1.78	5.49 ± 1.82
Present study	9.2 ± 1.09	6.97 ± 0.99

In this study, there were neither visceral nor vascular injuries reported in both methods. There was also no need for conversion to open cholecystectomy.

Juneja also reported similar findings where no major visceral/vascular injuries or gas embolisms occurred in any of the study groups [1]. Bonjer et al. reported that the rates of visceral injury were 0.08% and that of vascular injury was 0.07% with the closed method of pneumoperitoneum, whereas the rates of visceral injury was 0.05% and that of vascular injury were 0 % with the open method of pneumoperitoneum (p=0.002) [25].

Chapron et al. reported that the rate of bowel injury is 0.04% and that of major vessel injury is 0.01% in the closed technique of pneumoperitoneum, whereas the rate of bowel injury is 0.19% and that of major vessel injury is 0% in the open technique of pneumoperitoneum. They concluded that the open pneumoperitoneum method does not reduce the risk of complications during access [26]. Chandler et al. also observed that the open technique of pneumoperitoneum had no advantage over the closed technique when it comes to safety [27]. Jansen et al. observed that the rate of complications associated with the closed technique of pneumoperitoneum was 0.07%, and that of the open technique was 0.17%. The rate of entry-related complications was significantly higher with the open technique. Therefore, no evidence could be drawn to support the idea of abandoning the closed entry technique. Nevertheless, selecting patients is still recommended for an open or alternative procedure [10,28]. Meta-analysis failed to effectively demonstrate any safety advantage of an open over a closed method of entry regarding visceral and major vascular injury [14].

Our study did not observe post-op complications like port-site hematoma, port-site infection, or port-site hernia. Other studies would contradict these findings. Tariq et al. reported a 2.6% incidence of umbilical port site infection and a 1.3% incidence of port site hematoma with the open pneumoperitoneum method, whereas, in the closed group, no patient developed port site hematoma or infection. Other studies also suggested similar findings of port site infection in open groups. Den Hoed et al. reported the incidence of umbilical port-site infection to be 5.3%, Shindholimath et al. reported the incidence to be 6.3%, and Colizza et al. reported the incidence to be less than <2% [29,30]. No umbilical port site hernia was seen in either group at the follow-up of at least one month. 

The total operating time in our study was shorter in the open method group. This was probably due to less time taken to create pneumoperitoneum at the start and a faster rate of closure of wounds at completion. And also the routine performance of Veress needle entry tests like aspiration and saline tests in closed method groups. The time required to close the wounds in group A was more prolonged than in group B. In group B, the stay sutures applied to the margins of the sheath, in the beginning, facilitated the closure at a faster pace. In contrast, in group A, the facial margins at the umbilicus were difficult to keep hold of and take bites in depth through the small opening rendering it almost like a blind procedure.

Total hospital stay was slightly more in group A. However, these variables were statistically insignificant, including total operating time, the time required for wound closure and the entire hospital stay in either group.

## Conclusions

Laparoscopic cholecystectomy has become the gold standard in treating gallstone disease. The open technique for pneumoperitoneum is as safe and effective as the closed technique and is a good alternative to the closed technique. Further multicentric studies with larger sample sizes are required for more conclusive evidence.
